# A mutation in the *tuft* mouse disrupts TET1 activity and alters the expression of genes that are crucial for neural tube closure

**DOI:** 10.1242/dmm.024109

**Published:** 2016-05-01

**Authors:** Keith S. K. Fong, Robert B. Hufnagel, Vedbar S. Khadka, Michael J. Corley, Alika K. Maunakea, Ben Fogelgren, Zubair M. Ahmed, Scott Lozanoff

**Affiliations:** 1 Department of Anatomy, Biochemistry, and Physiology, John A. Burns School of Medicine, University of Hawai'i, Honolulu, HI 96813, USA; 2 Department of Pediatrics, Division of Human Genetics, Cincinnati Children's Hospital, College of Medicine, University of Cincinnati, 3333 Burnet Ave, ML 7003, Cincinnati, OH 45229, USA; 3 Unit on Pediatric, Development & Genetic Ophthalmology, Ophthalmic Genetics and Visual Function Branch, National Eye Institute, National Institutes of Health, Bethesda, MD 20892, USA; 4 Office of Biostatistics and Quantitative Health Sciences, John A. Burns School of Medicine, University of Hawai'i, Honolulu, HI 96813, USA; 5 Epigenomics Research Program, Department of Native Hawaiian Health, John A. Burns School of Medicine, University of Hawai'i, Honolulu, HI 96813, USA; 6 Department of Otorhinolaryngology Head and Neck Surgery, School of Medicine, University of Maryland, BioPark Bldg1, 800 West Baltimore Street, Room 404, Baltimore, MD 21201, USA

**Keywords:** Anterior cranial cephalocele, Midfacial cleft, Neural tube defect, Encephalocele, Exencephaly, Anencephaly, Epigenetic

## Abstract

Genetic variations affecting neural tube closure along the head result in malformations of the face and brain. Neural tube defects (NTDs) are among the most common birth defects in humans. We previously reported a mouse mutant called *tuft* that arose spontaneously in our wild-type 3H1 colony. Adult *tuft* mice present midline craniofacial malformations with or without an anterior cephalocele. In addition, affected embryos presented neural tube closure defects resulting in insufficient closure of the anterior neuropore or exencephaly. Here, through whole-genome sequencing, we identified a nonsense mutation in the *Tet1* gene, which encodes a methylcytosine dioxygenase (TET1), co-segregating with the *tuft* phenotype. This mutation resulted in premature termination that disrupts the catalytic domain that is involved in the demethylation of cytosine. We detected a significant loss of TET enzyme activity in the heads of *tuft* embryos that were homozygous for the mutation and had NTDs. RNA-Seq transcriptome analysis indicated that multiple gene pathways associated with neural tube closure were dysregulated in *tuft* embryo heads. Among them, the expressions of *Cecr2*, *Epha7* and *Grhl2* were significantly reduced in some embryos presenting neural tube closure defects, whereas one or more components of the non-canonical WNT signaling pathway mediating planar cell polarity and convergent extension were affected in others. We further show that the recombinant mutant TET1 protein was capable of entering the nucleus and affected the expression of endogenous *Grhl2* in IMCD-3 (inner medullary collecting duct) cells. These results indicate that TET1 is an epigenetic determinant for regulating genes that are crucial to closure of the anterior neural tube and its mutation has implications to craniofacial development, as presented by the *tuft* mouse.

## Abstract (precis)

**Summary:** We propose an epigenetic mechanism establishing the regulation of genes that are crucial for neural tube closure. This mechanism could be a novel target for resolving such birth defects and associated disorders.

## INTRODUCTION

Neural tube defects (NTDs), such as anencephaly, encephalocele and spina bifida, are among the most common birth defects in humans, with estimates of over 2650 annual cases in the United States (Centers for Disease Control; www.cdc.gov). Despite significant reductions, largely owing to prenatal supplementation of folic acid (FA) over the past 18 years in the United States (mandatory fortification authorized in 1996, but not fully implemented until 1998), NTDs remain among the most common serious birth defect. Furthermore, we still do not understand how FA prevents NTDs (reviewed in Copp et al., 2013). Some studies indicate that FA might have an adverse effect, depending on the individual's genetic background (Marean et al., 2011). There are over 450 loci in mice documented in the Mouse Genome Informatics (MGI) database that are associated with neural tube closure defects. This underscores the complexity of neurulation and the different ways by which closure defects might arise. Thus, alternative means for treatment and preventive care are necessary to address a broader spectrum of NTDs.

Strides have been made in understanding fundamental mechanisms of neural tube closure using various animal model systems. Gene mutations affecting components mediating planar cell polarity and convergent extension in mice associate with craniorachischisis, a severe and rare type of NTD in humans (Wallingford, 2012; De Marco et al., 2014; Murdoch et al., 2014). Environmental factors affecting epigenetic regulation of genes associated with neural tube closure have also been examined. Maternal diabetes and obesity, for example, are risk factors for NTDs. One of the largest studies to date indicated that the odds ratio of encephaloceles was more than threefold higher in infants born to diabetic mothers, and anencephaly was almost twofold more common (Garne et al., 2012). Studies report that the expression of genes associated with neural tube closure is significantly reduced in mouse models for diabetes by altering the distribution of modified histones (Salbaum and Kappen, 2010, 2012; Zhang et al., 2013) or methylation patterns of gene loci (Wei and Loeken, 2014; Wang et al., 2015) in the embryos of afflicted mothers. Furthermore, the embryo's chromatin landscape that primes gene expression could also be affected by the maternal diet (Salbaum and Kappen, 2010). Therefore, understanding the distribution of epigenetic marks in specific disease conditions will help us identify candidate genes that are potentially affected, and enable us to work towards approaches to prevent NTDs.

DNA methylation is a dynamic epigenetic mechanism for regulating gene expression, and is conserved in diverse species. DNA methyl transferases (DNMTs) catalyze the methylation of 5-cytosine of CpG dinucleotides to form 5-methylcytosine (5mC) throughout the mammalian genome (reviewed in Smith and Meissner, 2013). The methylated state of CpGs has pivotal roles in influencing gene transcription during embryonic development, imprinting, X-chromosome inactivation and cancer. Methylation states are dynamically reversed by TET (ten-eleven translocation) enzymes. TET enzymes catalyze the conversion of 5mC to 5-hydroxymethylcytosine (5hmC), thus subsequently producing a demethylated state of cytosines, and epigenetically determine which genes can be expressed in a given cell or tissue (Tahiliani et al., 2009). Recent findings indicate that 5hmC-mediated epigenetic regulation is crucial to neurodevelopment, aging and human diseases (Tan and Shi, 2012; Kaas et al., 2013; Pastor et al., 2013; Tsagaratou and Rao, 2013; Yamaguchi et al., 2013).

In mammals, there are three TET proteins, which are encoded by separate genes (*Tet1-3*). These proteins exhibit the same catalytic activity but are distinct in their expression levels and distribution at particular stages of development (Globisch et al., 2010; Ito et al., 2010; Szwagierczak et al., 2010; Gu et al., 2011; Yamaguchi et al., 2012). TET3 is primarily responsible for the global erasure of 5mCs in the paternal genome upon fertilization of the oocyte (Iqbal et al., 2011; Wossidlo et al., 2011) and in later stages of murine primordial germ cell (PGC) development to re-establish a pluripotent state (reviewed in Wu and Zhang, 2014). Without TET3, mouse embryos die during early embryogenesis (Gu et al., 2011). TET1 and TET2, however, seem to have more specific roles in establishing gene transcriptional programs by defining the genomic landscape in developing cell populations.

Gene knockouts for either *Tet1* or *Tet2* suggest that they can partially compensate the function of each other. Mice deficient in TET1 protein alone appeared normal and had no loss of progeny despite reductions in the level of 5hmC (Dawlaty et al., 2011). However, about a third of mice homozygous for the *Tet1^−/−^* knockout were smaller than normal. Some of them eventually grew to normal size and weight following 1 month in age. Mice deficient in TET2 alone were similarly viable, but about a third developed malignancies resembling myeloid leukemia, indicating the significance of TET2 in hematopoiesis (Ko et al., 2011; Li et al., 2011). Double-knockout mice deficient in both TET1 and TET2 developed to term, but many died and had severe gross abnormalities such as exencephaly, cranial hemorrhaging or growth retardation, indicating the role of these proteins during embryonic development (Dawlaty et al., 2013). How TET1 or TET2 affect the expression of genes associated with neural tube closure or cranial development is not yet known.

The *tuft* mouse presents traits resembling *Tet1*/*Tet2* double-knockout mice. Affected newborn mice present anterior facial malformations, resulting in a midfacial cleft, cranial cephalocele or both, to varying severity (Fong et al., 2012). However, the most severe defects are observed as early as embryonic day (E)8.5-9.0, during closure of the anterior part of the neural tube (Fong et al., 2014). Tips of the anterior folds fail to adhere and curled. These embryos likely resulted in exencephaly, which we also observed at E10.5 and later stages, or, to a lesser extent, incomplete closure of the anterior neuropore. Through whole-genome sequencing of the *tuft* mouse, we have identified a nonsense mutation within the *Tet1* gene that disrupts its catalytic function. RNA-Seq analysis and quantitative real-time PCR (qPCR) indicated that the mutation affected the expression of genes in multiple pathways associated with neural tube closure. We hypothesize that these defective TET1 proteins were able to bind their designated targets needed for neural tube closure and morphogenesis of the frontonasal region, but altered their expression. We propose an epigenetic mechanism for regulating closure of the anterior neural tube in the *tuft* mouse. *Tet1* is thus a candidate gene locus for predicting defects to neural tube closure and craniofacial development in humans.

## RESULTS

### Affected *tuft* mice were homozygous for a point mutation within the *Tet1* gene

The genomes of five mice that were family members exhibiting the *tuft* phenotype or carrying the affected allele, and one wild-type mouse of the same background strain, were sequenced. The family of mice consisted of an affected male that exhibited craniofacial malformations with a cephalocele, which we previously described as a *tuft* trait (Fong et al., 2012), a normal-appearing female predicted to be a carrier for the *tuft* allele and three of their pups. Two pups were affected, one with a cephalocele and the other with a midfacial cleft. The third pup did not seem to be affected. Therefore, the predicted genotype for mice with visible *tuft* traits was homozygous for the mutant allele (*tu*/*tu*) and the predicted genotype for normal-appearing carriers was heterozygous (*tu*/+).

**Fig. 1.**
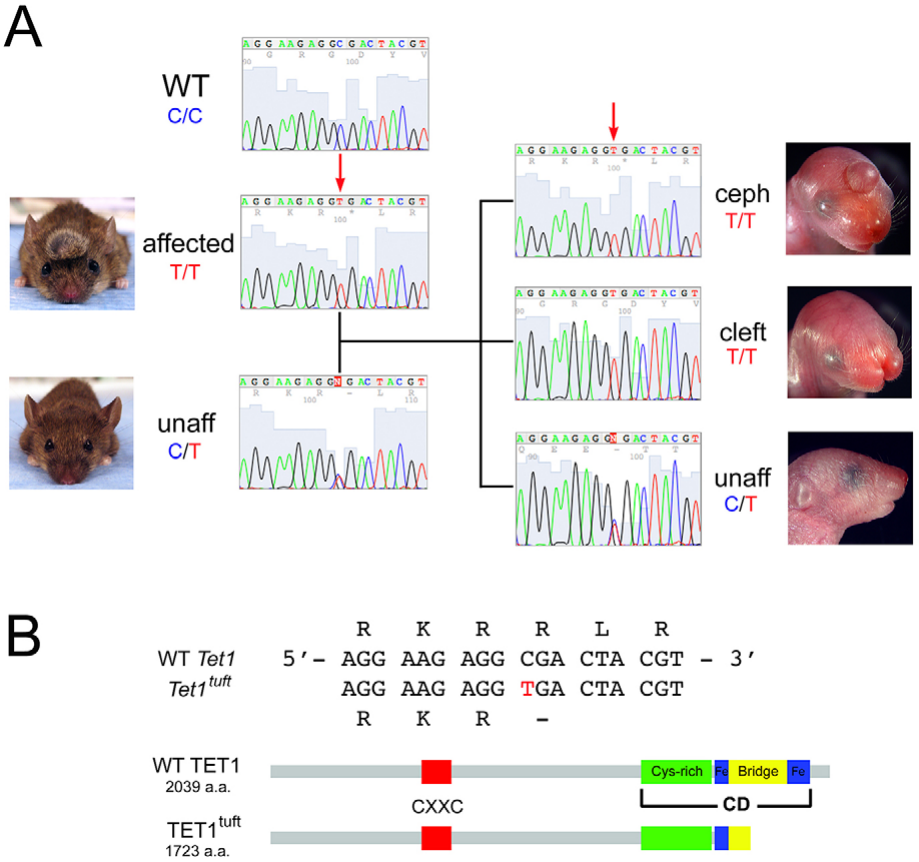
**Nonsense mutation in the *Tet1* gene of *tuft* mice.** (A) Example Sanger sequence showing mutation (arrow) from C in wild type (WT) or the 3H1 background strain to T in affected *tuft* mice with a cephalocele (ceph) or cleft. This mutation is carried by normal-appearing mice (unaff). (B) Sequence of *Tet1*, indicating that the C>T mutation results in a truncated TET1^tuft^ protein lacking the C-terminal iron-binding site (blue boxes) within the catalytic domain (CD). Domain structure adapted from Tan and Shi (2012).

Sequences were assembled and analyzed against the NCBI37/mm9 reference sequence from the University of California Santa Cruz (UCSC) Genome Bioinformatics database. A list of nucleotide variations based on the predicted genotypes of the mice was first obtained for chromosome (Chr) 10 because it was initially linked to the *tuft* phenotype (Fong et al., 2012). From a list of 72 variants on Chr 10, 43 were known strain-specific polymorphisms. Of the remaining variations, only one affected the coding sequence of a gene, *Tet1*. Compared to the reference sequence (MGI) and background strain mouse, a single cytosine was substituted by thymine in the first nucleotide position of a codon, encoding for arginine, in exon 11 (c.5167C>T), resulting in a termination codon (p.R1723*) (Fig. 1). This disrupted a potential nuclear localization signal sequence and excluded the last 318 amino acids of the catalytic domain (CD), thus likely rendering it non-functional. The His-x-Asp (HxD) motif, which coordinates with Fe(II) and is required for catalyzing 5mC to 5hmC (Tahiliani et al., 2009), remained intact.

We confirmed our genome analysis by Sanger sequencing the region containing the putative mutation in over 50 mice and embryos. These included samples that were used for whole-genome sequencing, some of the affected animals used for the initial linkage analysis (Fong et al., 2012) and others spanning generations including normal-appearing mice predicted to be carriers, and with other mice with phenotypes unrelated to *tuft* that were housed in the same facility serving as external negative controls. Nearly all affected mice (48/50) exhibiting one of the craniofacial traits from adults, newborns (ocular hypertelorism, cranial cephalocele, bifid nose, midfacial cleft or anencephaly) and embryos with neural tube closure defects that we previously described (Fong et al., 2014) were homozygous for the c.5167C>T mutation in the *Tet1* gene (*Tet1^tuft/tuft^*). Each of the other two mice had a very small cephalocele. We predicted these mice to be homozygous for the mutation but they were heterozygous, which might reflect a dominant effect. All normal-appearing predicted carriers we sampled (6/6) were heterozygous for cytosine and thymine at the same c.5167 position (*Tet1^tuft/+^*). Wild-type strains (4/4), including 3H1 (*tuft* background strain), BALB/c, C57BL/6J and a *Brachyrrhine* mouse housed in the same room with *tuft*, were homozygous for cytosine. The relative location of this mutation was consistent with our initial candidate region between 27 and 45 cM on Chr 10 (32.48 cM) predicted by linkage analysis (Fong et al., 2012). No other significant variations affecting the coding sequences (exons) of the mouse genomes were detected. Therefore, the mutation in *Tet1*, which we will refer to as *Tet1^tuft^*, is likely to be the primary genetic defect responsible for the *tuft* phenotypes.

### Reduced body size and TET activity in *Tet1^tuft^* mice

**Fig. 2.**
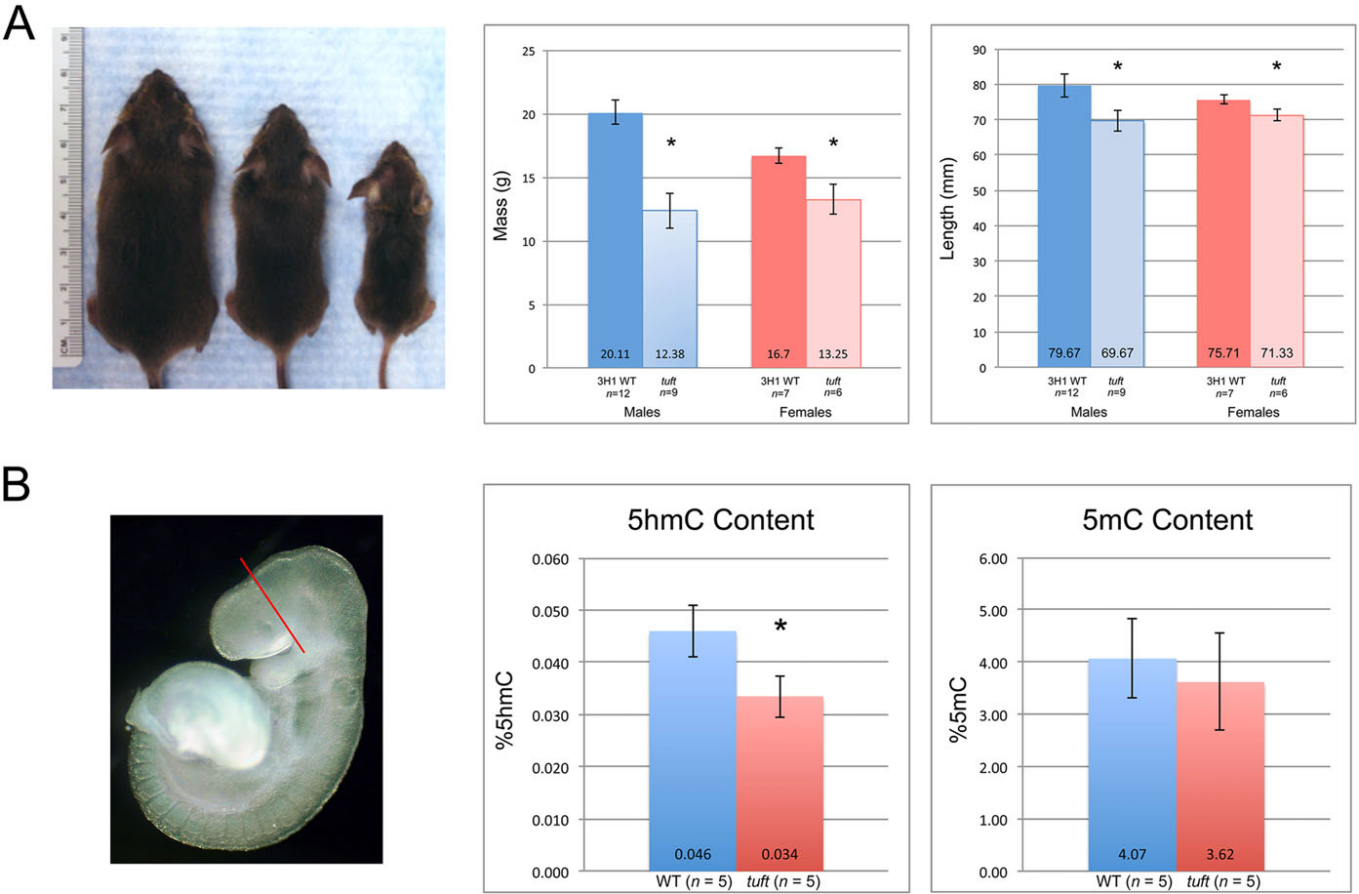
**Reductions in size and TET1 activity in *tuft* mice.** (A) The image shows 30-day-old mice: heterozygous for *Tet1^tuft^* and appearing normal (left), a homozygous *Tet1^tuft^* runt (middle) and an extreme case (right). Graphs show differences in body mass (grams) and length (millimeters) between 30-day-old runts and 3H1 Balb wild-type (WT) male and female mice. Mean values measured from *n* mice indicated at the base. Error bars indicate s.d. Significance determined by two-tailed unpaired Student's *t*-test indicated *P*<0.005 (*). (B) DNA was purified from dissected rostrums of E9 embryos demarcated by the red line. Graphs show the amount of 5hmC and 5mC in rostrums of E9 embryos homozygous for the *Tet1^tuft^* mutation compared with the 3H1 Balb wild-type background strain (WT). *P*=0.002 (*).

In light of the *Tet1^tuft^* mutation and phenotype of *Tet1* knockout mice, we noted that a significant number of pups from *tuft* matings were smaller than typical littermates or normal mice of the same age and sex (Fig. 2A). About 30% (28/96) of 30-day-old mice from *Tet1^tuft/tuft^*×*Tet1^tuft/+^* matings were reduced in body weight and length when 50% would be expected based on Mendelian inheritance for a recessive trait. This percentage might be an overestimate because mice that died *in utero* or were stillborn were not accounted. Male runts were almost 40% less than the average body weight of sex- and age-matched wild-type background mice or normal-appearing siblings (12.38 g±1.39 s.d., vs 20.11 g±0.93 s.d., *P*=0.0001). Their nose-to-rump length differed by less than 10% compared with wild-type (69.67 mm±3.08 s.d., vs 79.67 mm±3.28 s.d., *P*=0.00013). Female runts, on the other hand, weighed about 20% less than the normal body weight (13.25 g±1.20 s.d., vs 16.70 g±0.6 s.d., *P*=0.0011) and were just about 6% shorter in length (71.33 mm±1.63 s.d., vs 75.71 mm±1.25 s.d., *P*=0.0031). There were few extreme cases where mice weighed less than 10 g (*n*=5), or about 50% of the normal weight, at 1 month of age regardless of sex (shown in Fig. 2A but not included in the data set). Almost a third of runt mice (12/42) also exhibited one or more of the craniofacial traits (cranial cephalocele, hypertelorism, bifid nose) characteristic of the *tuft* traits that we previously described (Fong et al., 2012). But, like the craniofacial traits, the occurrence of the runt phenotype was lower than expected for complete penetrance. This observation is consistent with the phenotype in mice deficient in *Tet1*, with the exception of the developmental defects and lower penetrance. Genetic knockout mice for the *Tet1* gene were reported to be viable and fertile, but about 75% of homozygous mutant pups (13/17) had a smaller body size at birth (Dawlaty et al., 2011). Homozygous *Tet1* knockout pups averaged 8 g compared with 11 g, or about 27% less than normal. Some grew to normal body weight following 1 month in age as in the case for *tuft* runts.

We then sequenced the *Tet1* gene in mice with the runt phenotype with (*n*=6) or without (*n*=6) the *tuft* craniofacial traits. Runt mice that did not exhibit a craniofacial trait, hence reflecting the phenotype observed in *Tet1* knockout mice, were either homozygous (*n*=2/6) or heterozygous (*n*=4/6) for the same nonsense mutation, whereas runts that also presented one or more of the *tuft* craniofacial traits were always homozygous (*n*=6/6). This indicates a tight association between the craniofacial traits and homozygosity of the *Tet1^tuft^* mutation. Furthermore, the occurrence of a runt phenotype in mice heterozygous for the mutation suggests that it can have a dominant negative effect in *tuft* mice and partially prevent compensation by normal protein.

Because premature termination within the C-terminal domain would conceivably render the catalytic function of TET1 nonfunctional, we compared the amount of genomic 5hmC in *tuft* embryos that were homozygous for the mutation with corresponding tissues from the wild-type background strain. We found that the amount of genomic 5hmC in the anterior or rostral part of E9 embryos (18-22 somites) homozygous for the *Tet1^tuft^* allele was significantly less (0.034±0.004% s.d., *P*=0.002) than wild-type embryos (0.046±0.003% s.d.; 74% of normal) (Fig. 2B). This difference was not as large as what was observed in *Tet1* knockout mice (60% of normal), whereas mice deficient of both *Tet1* and *Tet2* had levels that were approximately 75% of normal (Dawlaty et al., 2011, 2013). This decline signified a loss of TET1 function in *tuft* embryos, which express a truncated protein. Furthermore, this loss could not be fully compensated by TET2 activity in *tuft* embryos.

We also examined the relative amount of genomic 5mC because we hypothesized that a reduction in TET1 activity would result in a similar or higher amount of methylated DNA than normal, as seen in mouse embryonic stem cells (mESCs) undergoing differentiation or depleted of TET1 (Ficz et al., 2011). We found that the amount of 5mC was not significantly different in E9 *Tet1^tuft^* embryo heads (3.62±0.93% s.d.) than in wild type (4.07±0.75% s.d.) (*P*=0.37). These values were comparable to what has previously been observed in *Tet1/Tet2* double-knockout mice, in which 4% of cytosines were methylated, compared with 3.5% in wild type (Dawlaty et al., 2013).

### Expression of *Tet1* RNA and protein in *tuft* embryos

**Fig. 3.**
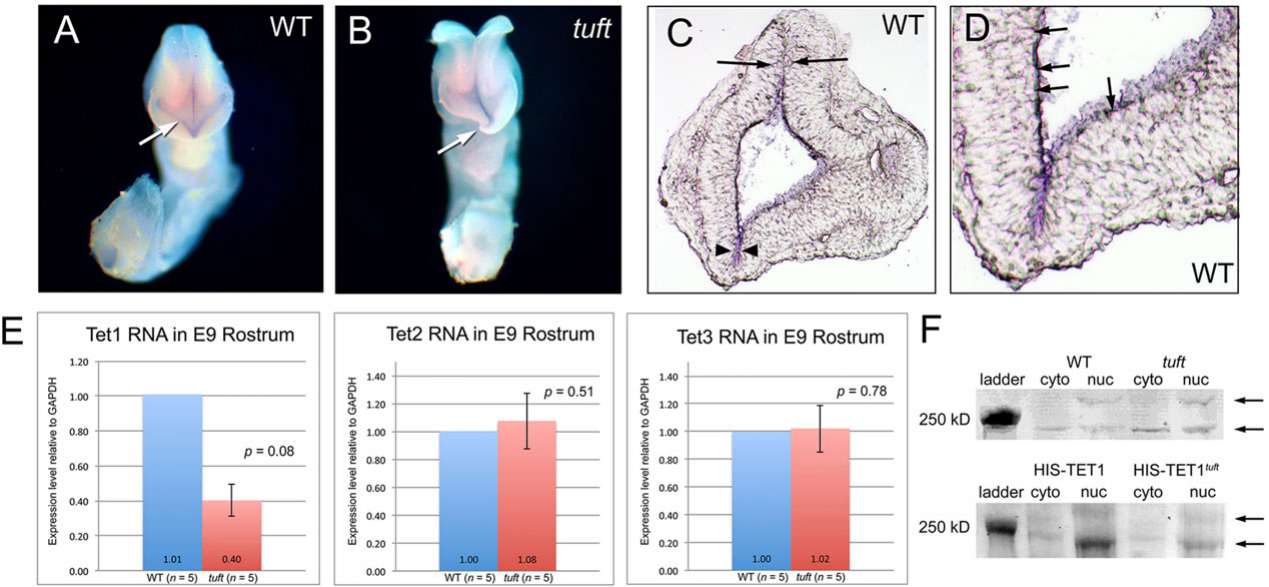
***Tet1*** **is** **expressed in neuroectoderm of E9 heads.** (A) Whole-mount staining revealed *Tet1* riboprobes (purple) along the neuroectoderm (arrow) of E9 wild-type (WT) and (B) *Tet1^tuft^* embryos. Curled rostrum (arrow), open midbrain and less intense purple staining along the basal neuroectoderm is noted in the *Tet1^tuft^* mutant. (C) Coronal section of a WT embryo head stained for *Tet1* riboprobe as shown in A, indicating expression along the ventral (arrowheads) and dorsal (arrows) midline, where the neural folds adhere, enclosing the neuroectoderm. (D) Magnified area of the ventral region shown in C, indicating stained basal cells along the neuroectoderm (arrows). (E) Fold-change differences of *Tet1*, *Tet2* and *Tet3* RNA in *Tet1^tuft^* E9 rostrums compared with 3H1 Balb (WT), normalized to GAPDH levels. Mean values of biological replicates (*n*) are shown at the base with error bars marking s.d. Statistical significance is indicated as *P*-values. (F) Western blots indicating TET1 protein (arrows) in cytoplasmic (cyto) and nuclear (nuc) fractions in wild-type (WT) and *Tet1^tuft^* mice near the 250 kD marker. Lower blot indicates recombinant poly-histidine (HIS)-tagged TET1 and TET1^tuft^ protein expressed in IMCD-3 cells (arrows).

Because the *Tet1^tuft^* point mutation resulted in premature termination, we wanted to determine whether *Tet1* mRNA was still present and producing protein in *tuft* embryos. We compared the expression of *Tet1* in *tuft* embryos with its wild-type 3H1 Balb background strain by whole-mount *in situ* hybridization (WMISH) and qPCR. The expression of *Tet1* was most prominent in cells along the apical side of the neuroectoderm facing the ventricular space and along the dorsal midline in normal E9.0-9.5 (14-24 somites) embryonic heads by WMISH (Fig. 3A-D). This was consistent with what was observed in E9 embryos containing a *Tet1*-*lacZ* genetrap construct (Yamaguchi et al., 2012). Staining in *tuft* embryos with neural tube closure defects was similar in localization, although somewhat less intense (Fig. 3B). We then analyzed the expression of *Tet1* RNA in the rostral part of the head in embryos from different stages during and following closure of the anterior neural tube (14-24 somites) by qPCR. The amount of *Tet1* RNA from E9 *Tet1^tuft^* embryos homozygous for the mutation was less than half of what was measured in wild-type embryos of a similar stage (Fig. 3E). Levels of *Tet2* and *Tet3* RNA were not significantly different from wild-type embryos (*P*=0.57 and 0.78, respectively).

We assessed whether mutant protein was still present or degraded in *tuft* mice, despite the reduced levels of RNA. Western blots using an antibody specific to the N-terminal region of TET1 detected bands approximating the predicted molecular weights in tissue samples from both 3H1 wild-type and *tuft* mice homozygous for the *Tet1* mutation (Fig. 3F). Two bands appeared in the nuclear fraction flanking the 250 kD molecular mass marker. The band corresponding to the lower molecular mass was only present in the cytoplasmic fraction. These bands were larger than the calculated molecular masses of the two alternatively spliced TET1 variants (220 and 200 kD). The band larger than the 250 kD marker in our western blots was similarly observed by others (Jin et al., 2014), whereas the lower band was similar to that seen in mESCs (Dawlaty et al., 2011). Because none of these reported bands corresponded exactly to the predicted molecular masses, it is likely that they are post-translationally modified forms. The predicted molecular mass of the truncated TET1^tuft^ protein, however, is about 190 kD. We could not clearly resolve this difference from wild type no matter how far samples were run through the gel. To verify that these two bands corresponded to TET1, we overexpressed recombinant TET1 and mutant TET1^tuft^ protein fused to a polyhistidine (poly-HIS) epitope tag in IMCD-3 (inner medullary collecting duct) cells. Two bands of similar molecular masses as seen in our tissue samples were also detected using antibodies against either poly-HIS epitope tag (lower blot in Fig. 3F) or TET1. These bands were also visible in Coomassie-stained gels, but not in cells transfected with the control plasmid expressing just poly-HIS (not shown). These results indicate that TET1 protein is expressed and present to some degree in *tuft* mice homozygous for the mutation.

### Gene expression associated with anterior neural tube closure was altered in *Tet1^tuft^* E9 heads

The neural tube closure defects we observed in *tuft* embryos homozygous for the mutation suggests that they were affected by reduced TET1 protein or function. Neural tube closure defects have not been reported in single-knockout mice deficient in either *Tet1*, *Tet2* or *Tet3* (Dawlaty et al., 2011; Gu et al., 2011; Ko et al., 2011; Li et al., 2011). However, exencephaly and cranial defects were reported in *Tet1/Tet2* double-knockout mice (Dawlaty et al., 2013). We wanted to determine how the truncated TET1^tuft^ protein resulted in the neural tube closure defects that we observed in *tuft* embryos.

Because TET1 was shown to affect gene transcription in mESCs (Williams et al., 2011; Wu et al., 2011), we wanted to determine whether genes associated with neural tube closure were affected in *tuft* embryos. We compared the levels of RNA from the anterior part of E9 (16-22 somites) *Tet1^tuft^* heads that presented curled neural folds with corresponding 3H1 Balb wild-type background strain embryos by RNA-Seq. Following statistical analysis, RNA levels from 2957 of the 24,487 genes identified (12.08%) were significantly different (*q*<0.05) compared to wild type. In total, 1530 of the 2957 genes were downregulated (51.7%), whereas 1427 genes were elevated (48.3%). The expression of 104/465 gene loci (22%) associated with neural tube closure defects listed in the MGI database for mouse phenotypes (MP:0002151) was significantly altered (*q*<0.05). About 45% of those (47/104) were associated with TET1 binding in mESCs based on the data from Williams et al. (2011). A total of 30 of those genes were downregulated (64%), whereas 17 remained elevated (36%). Among those 465 genes that were listed in MP:0002151, 65 were also associated with incomplete closure of the rostrum or anterior neuropore as listed in MP:0000928. Expression was significantly changed in 14/65 of those genes (21.5%) in *tuft* embryos. We conducted similar surveys with genes associated with cellular adhesion, because that process seemed to be affected in *Tet1^tuft^* embryos. From these surveys, altogether, we noticed that a number of genes associated with the noncanonical wingless (WNT) signaling pathway were significantly dysregulated (*P_NOI_*>0.95) in *Tet1^tuft^* embryo heads (Table 1).

**Table 1.**
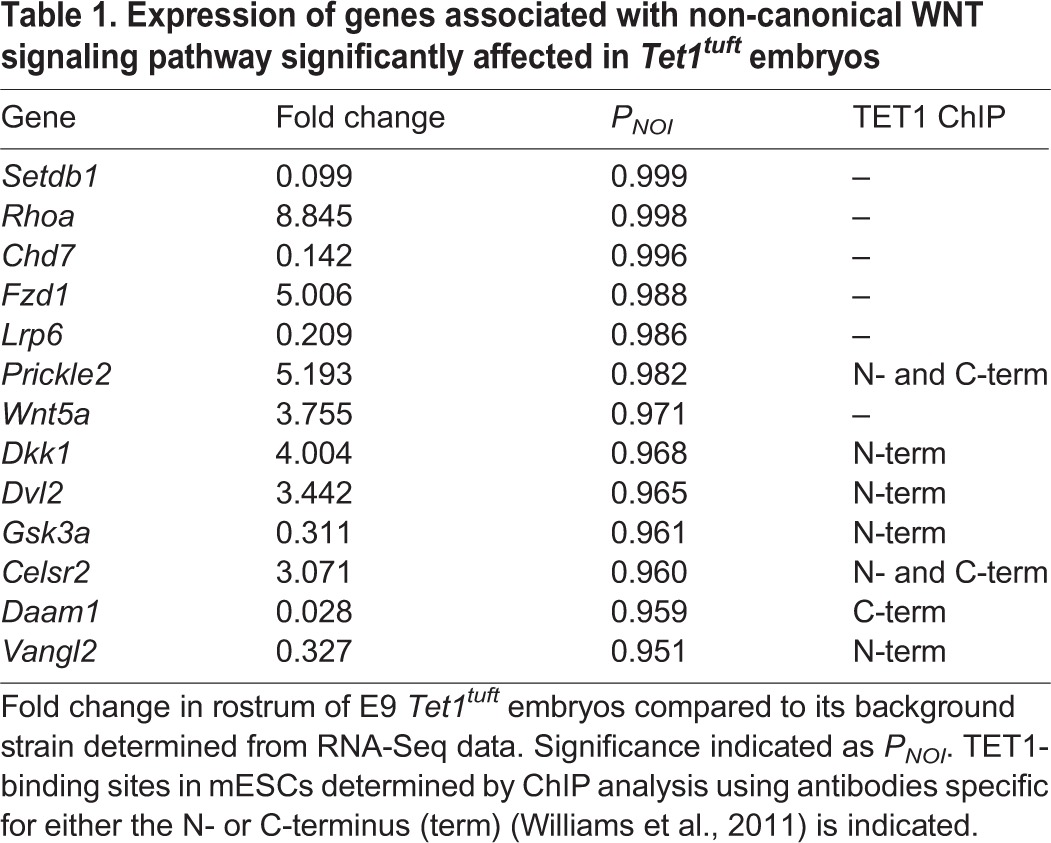
**Expression of genes associated with non-canonical WNT signaling pathway significantly affected in *Tet1^tuft^* embryos**

**Fig. 4.**
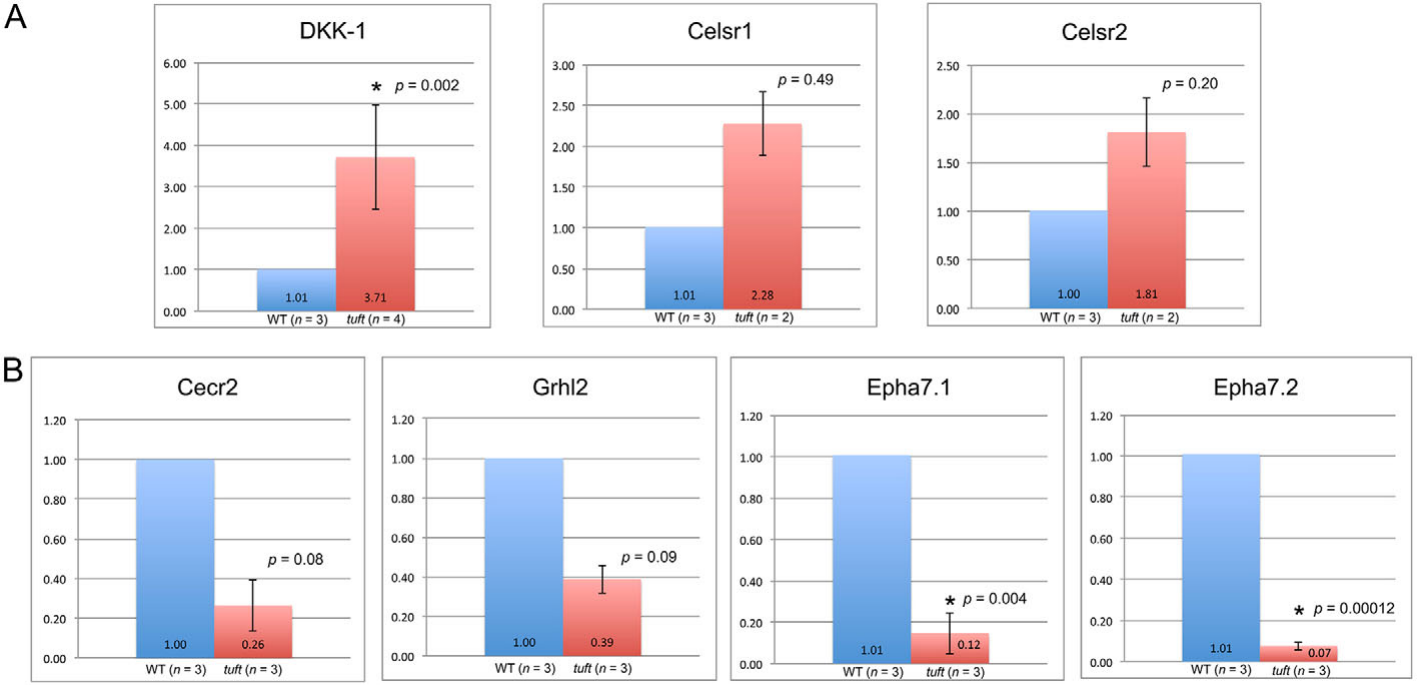
**Levels of RNA transcribed from genes associated with planar cell polarity and neural tube closure are altered in E9 *Tet1^tuft^* embryos.** (A) Fold changes in RNA levels of *Dkk1*, *Celsr1* and *Celsr2* from *Tet1^tuft^* embryo rostrums normalized against corresponding wild type (WT) relative to GAPDH by qPCR. Mean values of biological replicates (*n*) indicated at the base with error bars marking s.d. (B) Fold changes in RNA levels of *Cecr2*, *Grhl2*, *Epha7.1* and *Epha7.2* as in A. Statistically significant difference when *P*<0.05 indicated (*).

Considering that the initial RNA template used for analysis was pooled from three embryos bearing the same genotype, similar phenotype and age, we then analyzed the expression of several genes in individual samples by qPCR, particularly those with putative TET1-binding sites. We found that *Dkk1* expression was significantly elevated (*P*=0.002) in the anterior rostrum from a number of *tuft* E9 embryos (16-22 somites) with neural tube closure defects (4/6 affected embryos) compared with wild-type embryos of a similar stage (Fig. 4A), as indicated by our initial RNA-Seq analysis, whereas no significant change was detected in other affected *tuft* embryos (2/6). We also found that expression of either *Celsr1* (2/6 embryos) or *Celsr2* (2/6) was elevated in some of those same embryos with elevated *Dkk1*, even though we did not detect a statistically significant change in the expression of *Celsr1* from our initial RNA-Seq samples (*P_NOI_*=0.818, 0.592-fold change) based on our criteria. We were not able to detect consistent changes in the expression of *Dvl2*, *Daam1*, *Prickle2* and *Vangl2* from individual embryos by qPCR (data not shown), as indicated by our RNA-Seq results. Therefore, we could not firmly conclude whether WNT signaling was significantly affected.

Some embryos, however, did not indicate changes in the levels of *Celsr1* and *Celsr2*, but had reductions in the expression of *Cecr2* (cat eye chromosome region candidate 2) or *Grhl2* (grainyhead-like 2). Mice with mutations in either the *Cecr2* or *Grhl2* genes presented neural tube closure defects similar to *tuft* mice (Banting et al., 2005; Pyrgaki et al., 2011). Putative binding sites for TET1 were also detected in the promoter region in each of these genes (Williams et al., 2011). The expression of *Cecr2* and *Grhl2* declined following anterior closure of the neural tube in wild-type embryos (about 16-18 somites) but was lower in 3/6 *tuft* embryos of a comparable stage (Fig. 4B). We further found that the expressions of both ephrin receptor alpha 7 (*Epha7*) transcript variants were nearly depleted in these embryos. *Epha7* is a putative downstream target of CECR2 (Fairbridge et al., 2010) that is crucial for closure of the anterior part of the neural tube (Holmberg et al., 2000).

### *Grhl2* expression was reduced by TET1^tuft^ protein in cell culture

**Fig. 5.**
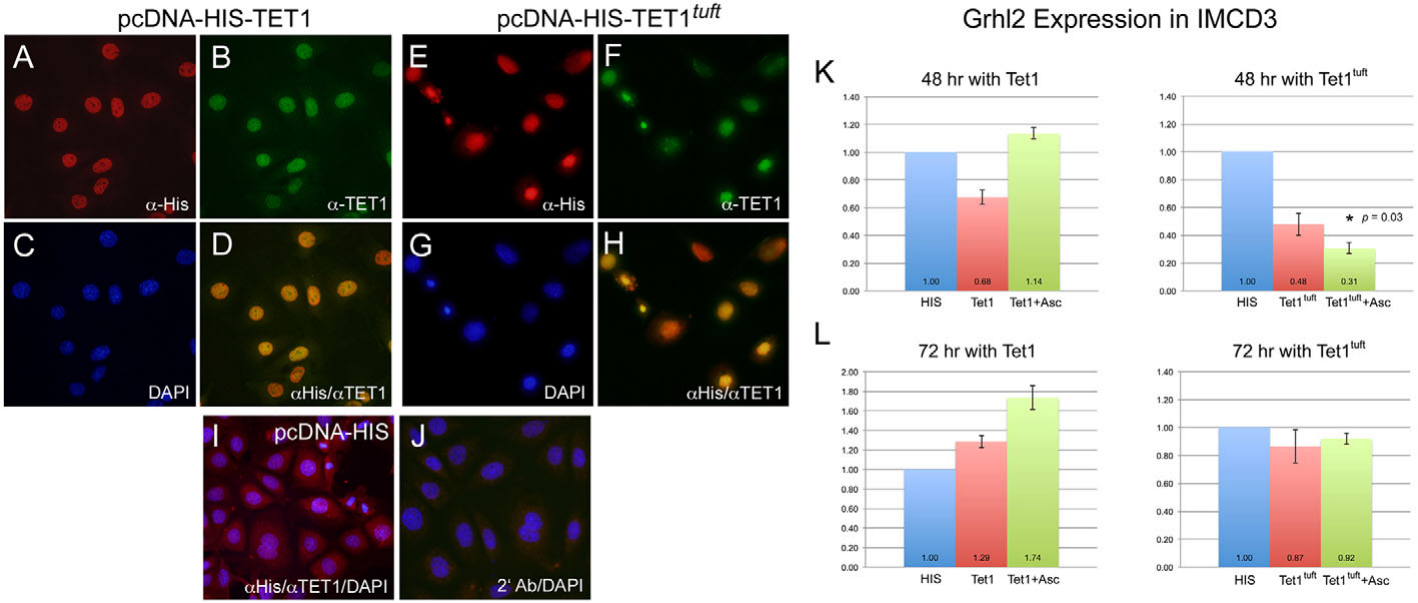
**Recombinant TET1^tuft^ protein reduced *Grhl2* RNA in IMCD-3 cells.** (A-D) Immunocytochemical localization of HIS-TET1 and (E-H) HIS-TET1^tuft^ recombinant protein overexpressed in IMCD-3 cells. (A,E) Cells stained for the anti-poly-HIS epitope tag (red), (B,F) the N-terminal part of TET1 (green), and (C,G) DAPI to reveal nuclei (blue). (D,H) Merged image colocalizing anti-HIS and anti-TET1 to nuclei (yellow). (I) IMCD-3 cells transfected with pcDNA-HIS vector stained for anti-HIS, anti-TET1 and DAPI. (J) *HIS-Tet1^tuft^*- transfected cells incubated with just secondary antibodies stained for all three colors. (K,L) Expression levels of *Grhl2* in IMCD-3 cells transfected with pcDNA-HIS vector (HIS), pcDNA-HIS-Tet1 (Tet1) or pcDNA-HIS-Tet1^tuft^ (Tet1^tuft^) with and without ascorbate (Asc) cultured for (K) 48 and (L) 72 h, relative to GAPDH. Mean values of replicates indicated at the base with error bars marking s.d. *P*=0.03 (*).

To determine whether the mutant TET1^tuft^ protein had an effect on gene expression, we overexpressed wild-type TET1 and mutant TET1^tuft^ recombinant protein in murine IMCD-3 cells that were known to express appreciable levels of *Grhl2* (Werth et al., 2010). We first determined whether the mutant TET1^tuft^ recombinant protein was capable of translocating into the nucleus, because the mutation disrupted a putative nuclear bipartite localization signal sequence in the linker region of the catalytic domain. IMCD-3 cells expressing either TET1 or TET1^tuft^ recombinant protein fused with a poly-HIS epitope tag at the N-terminus were primarily detected in the nucleus within 48 h from transfection (Fig. 5A-H). Cells transfected with the control plasmid expressing just the poly-HIS tag were positive for histidine immunostaining generally throughout the cell but negative for the TET1 antibody (Fig. 5I). Transfected cells incubated with just the secondary antibodies were also negative (Fig. 5J).

We monitored the expression levels of endogenous *Grhl2* in cells of similar confluence transfected with poly-HIS-tagged TET1 or TET1^tuft^ mutant protein, with or without the addition of ascorbic acid, for up to 72 hrs. Ascorbic acid is a co-substrate for TET enzyme activity (Blaschke et al., 2013; Yin et al., 2013). Levels of *Grhl2* RNA did not significantly change following 48 h of overexpressing TET1 compared with cultures transfected with the empty pcDNA-HIS vector as a negative control with or without ascorbic acid (Fig. 5K). There was a slight elevation of *Grhl2* transcription when comparing cultures supplemented with ascorbic acid. Cultures transfected with the mutant TET1^tuft^ recombinant protein, however, showed reductions in *Grhl2* expression, with (*P*=0.03) or without (*P*=0.15) the addition of ascorbic acid. After 72 h, the expression of *Grhl2* was elevated in cultures expressing wild-type TET1, especially in those supplemented with ascorbic acid (*P*=0.14, Fig. 5L). However, little or no change in expression was detected in the presence of mutant protein at 72 h (*P*=0.66). Levels of *Grhl2* in control cultures at 72 h were much lower than that detected at 48 h with or without ascorbate (data not shown). So, levels of *Grhl2* seemed to remain low in 72-h cultures with mutant TET1^tuft^ protein, but could be elevated in the presence of recombinant TET1 protein and ascorbate. These results are consistent with the observation that *tuft* embryos presenting neural tube closure defects have reductions in *Grhl2* RNA. This further supports that mutant TET1^tuft^ protein can exert a dominant negative effect by inhibiting gene transcription.

## DISCUSSION

Animal model studies have unraveled a large number of genes associated with neural tube closure and underscore the complexity of this process (Harris and Juriloff, 2007, 2010). Although gene mutations associated with neural tube closure in humans are being identified, there is a need for understanding how environmental conditions influence their expression in order to address a broader spectrum of cases. It has been shown, for example, that maternal conditions can dictate cellular programs for gene expression by defining the chromatin landscape. Altered distributions of modified histones (Pavlinkova et al., 2009; Salbaum and Kappen, 2010, 2012; Kappen et al., 2011) or methylated CpGs (Ichi et al., 2010; Zhang et al., 2013; Wei and Loeken, 2014; Wang et al., 2015) have been shown to directly affect genes associated with neural tube closure. Because these marks target multiple genes involved in neural tube closure, the genes affected might differ in a particular environment. Furthermore, their occurrence might not be carried over generations if these epigenetic marks are not maintained. This could account for the sporadic occurrence of specific NTDs and other birth defects within a family history. Variance will also depend on the maternal condition prior to and during pregnancy. Thus, NTDs might not only arise from mutations in genes directly involved with the mechanics of closure. We will need to know how epigenetic factors govern the organization of chromatin in order for us to understand how gene expression can be manipulated and alter the functionality of cells.

We previously found a heritable mutation disrupting the catalytic function of TET1 in the *tuft* mouse, which presents defects in neural tube closure or craniofacial development. These defects are generally restricted to the anterior midline or rostral part of the neural tube but vary in severity (Fong et al., 2012, 2014). Mice homozygous for the mutation could also exhibit one or more traits such as a midfacial cleft, cranial cephalocele and smaller body mass. Despite the reduced level of enzymatic activity, the mutation does not necessarily have a null effect, as in *Tet1* knockout mice (Dawlaty et al., 2011), but causes gross defects resembling mice that were deficient in both TET1 and TET2 (Dawlaty et al., 2013). The traits in *tuft* mice, however, cannot be completely attributed to a double null of TET1 and TET2. *Tet1^tuft^* RNA and protein was present in *tuft* mice homozygous for the mutation. We did not detect a significant difference in the expression of *Tet2* or *Tet3* from our RNA-Seq and qPCRs, or mutation affecting their coding region from genomic sequence analysis (data not shown). Thus, normal amounts of TET2 and TET3 were likely present and active in *Tet1^tuft^* mice, although unable to fully compensate for the loss of TET1 activity. Therefore, mutant TET1^tuft^ protein was present and interfered with compensatory activity, thus having a dominant negative effect. However, it is also possible that mutant TET1^tuft^ protein formed ectopic interactions with other molecules to generate a neomorphic trait, as in cases when a lipomatous cephalocele was formed. The presence of a lipomatous cephalocele in *Tet1/Tet2* double knockouts was not reported.

**Fig. 6.**
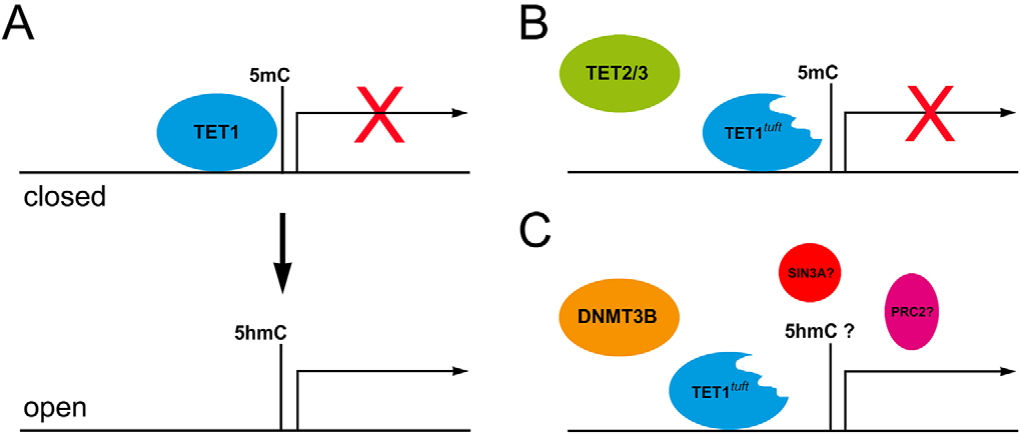
**Modeling TET1 function and the dominant negative effect of TET1^tuft^ protein.** (A) TET1 normally associates with 5mC, and gene transcription is generally silent or closed. Transcriptional complexes open following catalysis of 5mC to 5hmC by TET1, allowing for regulation. (B) In the case of *tuft* mice, TET1^tuft^ binds to target sites but is unable to catalyze conversion of 5mC to 5hmC, thus permitting transcriptional repression. Presence also interferes with compensation from TET2 or TET3 activity. (C) TET1^tuft^ might also be unable to interact with repressor complexes (SIN3A, PRC2) or allow methyltransferase activity (DNMT3B) to suppress gene transcription.

The truncated TET1^tuft^ protein interfered with the expression of genes associated with neural tube closure in *tuft* mice. This is consistent with the role of its C-terminal catalytic domain in regulating gene transcription. Because the methylation of gene promoters is typically associated with transcriptional repression, TET1 can promote transcription by catalytically removing 5mCs and preventing remethylation (Fig. 6A). Without this function, we would expect that genes regulated in this way might remain repressed in *tuft* mice. Indeed we found significant reductions in the amount of 5hmC and the expression of genes associated with the *tuft* phenotype, and thus TET1 activity. Many of the genes associated with neural tube closure were associated with TET1 binding based on chromatin immunoprecipitations (ChIPs) from mESCs (Williams et al., 2011). However, the expressions of all the genes with putative TET1-binding sites were not necessarily affected in each *tuft* embryo homozygous for the mutation presenting an NTD. Some embryos, for example, were deficient in *Cecr2* but others were not and instead were affected by dysregulated components of the noncanonical WNT signaling pathway. We suspect that mutant TET1^tuft^ protein was present, but insufficient to bind every site, possibly owing to degradation and partial compensation by TET2 or TET3.

Mice deficient in *Cecr2* closely resemble the traits of newborn *tuft* mice. Mice deficient in *Cecr2* primarily exhibit exencephaly (Banting et al., 2005) but also present a midline facial cleft with exencephaly or forebrain cephalocele in a different genetic background (Fairbridge et al., 2010). CECR2 is a chromatin modifier that heterodimerizes with SNF2L (mouse SMARCA1) to form CERF (CECR2-containing remodeling factor) (Banting et al., 2005). CERF modifies the position of nucleosomes in an ATP-dependent manner, thus altering the accessibility of transcriptional regulatory sites. *Cecr2* seems to be expressed throughout the mouse embryo, but prominently at the margins of neural folds during closure in E9 and in neural tissue through E13.5 (Banting et al., 2005). Disruption of *Cecr2* leads to a significant reduction in levels of *Alx1* (*Cart1*), *Dlx5* and *Epha7* RNA (Fairbridge et al., 2010). Mice deficient in any one of these genes have neural tube closure defects (Zhao et al., 1996; Acampora et al., 1999; Depew et al., 1999; Holmberg et al., 2000). Consistent with this finding, the expression of both *Epha7* isoforms was nearly depleted in the same *tuft* embryos with reduced levels of *Cecr2*. Therefore, closure was likely prohibited by the lack of *Epha7* through deregulation of *Cecr2* by mutant TET1^tuft^ protein in these cases. We hypothesize that the TET1^tuft^ protein was unable to catalytically reverse the repressive methylated state of the *Cecr2* promoter and its presence prohibited compensatory efforts by TET2 or TET3 (Fig. 6B). We demonstrated that mutant TET1^tuft^ protein could still enter the nucleus and deregulate the expression of *Grhl2* in IMCD-3 cells. However, not all genes associated with neural tube closure were suppressed in *Tet1^tuft^* embryos. This indicates that a mechanism for transcriptional repression could also be disrupted.

*Tet1^tuft^* embryos that did not have significant changes in *Cecr2* usually had abnormal expression levels in one or more components of the planar cell polarity proteins that mediate convergent extension. Deficiencies to one of these components in mice typically led to craniorachischisis, the most severe type of NTD involving caudal closure, but also a range of neural tube closure defects (Murdoch et al., 2014). Mutations have also been linked to human cases with NTDs, including lipomas (De Marco et al., 2014). Thus, we were somewhat surprised to find that the expression in one or more of these components remained elevated in *Tet1^tuft^* embryos with neural tube closure defects. The anterior neural folds of these embryos curled inward but did not appear to have problems with closure along the caudal portion of the neural tube as in craniorachischisis (Fong et al., 2014). CELSR1 and CELSR2 are protocadherin transmembrane adhesion proteins that are both expressed in the neural ectoderm during neural tube closure and brain development (Formstone and Little, 2001). CELSR1 recruits PDZ-RhoGEF, RhoA and Rho kinase (ROCK1) through interactions with DVL and DAAM1 to promote mediolateral contraction along the floor plate to bend the neural tube (Nishimura et al., 2012). Abnormally high levels of *Celsr1* could have prompted the elevated levels of *RhoA* that we detected in our initial RNA-Seq analysis, thus excessive actin-myosin contractile activity resulted in exaggerated curling of the neural folds. Whether abnormal expression levels of *Celsr2* affects neural tube closure is unclear. Mice deficient in *Celsr2* impaired ciliogenesis in ependymal cells, resulting in hydrocephalus (Tissir et al., 2010). *Celsr2* is necessary for correct positioning of cilia at the apical surface of ependymal cells. Elevated levels of *Celsr2* might result in an imbalance of CELSR-CELSR interactions, thus altering cell polarity or cellular organization. Because TET1 associates with the promoters of *Celsr1* and *Celsr2* (Williams et al., 2011) and other chromatin modifying factors (Wu et al., 2011; Cartron et al., 2013), elevated expression indicates that the truncated TET1^tuft^ protein lost its ability to repress transcription of these genes (Fig. 6C).

The significance of elevated *Dkk1* (dickkopf1) in *tuft* embryos with neural tube closure defects is not clear, especially because the expression of *Cecr2*, *Grhl2* or PCP genes could also have been affected in the same embryos. DKK1 is the major factor for anterior identity and head formation (Mukhopadhyay et al., 2001). Normal expression of *Dkk1* in the anterior region is thought to prevent the formation of neural crest cells by antagonizing canonical WNT signaling (Fossat et al., 2011). Thereby, anterior structures are contributed by cranial neural crest cells (CNCCs) emigrating from the midbrain region. It is possible that extended expression of *Dkk1* in *tuft* embryos prevented an adequate amount of CNCCs migrating toward the frontonasal region to enable normal craniofacial development. TET1 has been associated with *Dkk1* in mESCs (Williams et al., 2011). As in the case with elevated *Celsr1/2*, mutant TET1*^tuft^* protein might prevent timely downregulation of *Dkk1* during craniofacial development. Disruptions to WNT signaling by elevated *Dkk1* might account for craniofacial anomalies in *tuft* mice.

NTDs or craniofacial anomalies associated with mutations to *Tet1* have not been reported thus far. We focused our investigation on genes that are crucial to neural tube closure that are putative targets for TET1 based on the normal state of mESCs. Because TET2 or TET3 could not fully compensate the loss of activity in *tuft* embryos, it is likely that these genetic loci involved with neural tube closure were targets for TET1 activity. If so, we might be able to reduce or eliminate the negative effect of dysfunctional TET1^tuft^ protein and allow TET2 or TET3 to compensate its function. Alternatively, we could overcome the effects of dysfunctional TET1 protein by overexpressing normal enzyme or possibly augment TET activity by modifying the parental diet. We demonstrated that TET1 could promote the transcription of *Grhl2* in IMCD-3 cells supplemented with ascorbic acid, but was deregulated in the presence of mutant TET1^tuft^ protein. Variable activity owing to genetic background or dietary intake might dictate particular traits, physical or even mental, because TET1 is thought to play substantial roles in neural development and behavior. Mice deficient in TET1 alone have deficits in memory extinction, a behavioral mechanism allowing adaptation that might be impaired in cases of post-traumatic stress disorder (Rudenko et al., 2013). Heritable variants altering the function of TET1 in humans might result in such defects. It would be of value to know when TET1 is essential to elicit critical steps. The *tuft* mouse can thus serve as a model system to assess therapeutic strategies addressing NTDs and TET1-associated disorders. We must consider, however, that epigenetic marks regulating the same genes in humans could differ from mice or even between individuals. This might explain the variance in phenotype between species and susceptibility of disease.

## MATERIALS AND METHODS

### Animals

All procedures were carried out in accordance with Institutional Animal Care and Use Committee (IACUC) specifications and approved by the University of Hawai'i Laboratory of Animal Services. Mice strains were housed under standard conditions and bred as previously described (Fong et al., 2012). Timed matings were determined by noting the presence of a vaginal plug as day 0.5 for staging embryo collections and estimating date of births. Developmental stages of embryos were determined based on the number of somites. Genotyping of DNA isolated from tail clips or embryonic tissue was performed by PCR (primers: Forward 5′-GGTAGAACAGGCCTTATTCCTC-3′, Reverse 5′-GGTGAGAAGTAGATGAGGCTG-3′) followed by Sanger sequencing (sequencing primer: 5′-GGATGAACAACTCCACGTCCTG-3′).

### Library preparation and DNA sequencing

1 µg of double-stranded DNA (dsDNA), determined by using the Invitrogen sQbit (Life Technologies, Carlsbad, CA) high-sensitivity spectrofluorometric measurement, was sheared by sonication to an average size of 200 bp on a Covaris S2 instrument (Covaris, Woburn, MA). Library construction was performed in an automated fashion on an IntegenX Apollo324 (IntegenX, Pleasanton, CA) which size-selects fragments by double-SPRI (solid phase reversible immobilization) binding with different concentrations of polyethylene glycol (PEG) for a high cut and a low cut. Each library was fitted with an adapter containing a different six-base molecular barcode for high-level multiplexing. After nine cycles of PCR amplification using the Clontech Advantage II kit (Clontech Laboratories, Mountain View, CA), 1 µg of genomic library was recovered for genome enrichment using the PE125 kit. Libraries were enriched according to the manufacturer's recommendations and sequenced on an Illumina HiSeq2500 (Illumina, San Diego, CA), generating around 30-million 100-bp paired-end reads each equivalent to 6 GB of usable high-quality sequence per sample.

### Data alignment and analysis methods

Analysis methods utilized the Broad Institute's Genome Analysis Toolkit (GATK) and followed the pipeline described by DePristo et al. (2011), along with the modifications listed in the ‘Best Practices’ document on their website (http://www.broadinstitute.org/gsa/wiki/index.php/The_Genome_Analysis_Toolkit).

Briefly, reads that passed Illumina's Chastity Filter were aligned with the Burrows Wheeler Aligner (BWA) (Li and Durbin, 2009). GATK's UnifiedGenotyper module was used to call variant sites (both single nucleotide and small indel) in all samples simultaneously. Finally, single-nucleotide variant (SNV) calls were filtered using the variant quality score recalibration method described previously (DePristo et al., 2011). Indel calls were filtered with a set of hard filters because there are not enough indels in an exome to use the Gaussian method.

Variants were filtered using Golden Helix SNP & Variation Suite (SVS; www.GoldenHelix.com). Variants were filtered for quality >20. Variants from Chr 10 and matching segregation through expected genotypes were isolated and analyzed for pathogenicity and known frequency in healthy populations.

### TET activity assay

TET1 activity was indirectly estimated using the Quest 5-hmC and 5-mC DNA ELISA kits (Zymo Research, Irvine, CA). 200 ng of genomic DNA purified from the anterior part of E9 heads (14-22 somites) from *tuft* litters with NTDs and 3H1 Balb wild-type background embryos was measured for 5-mC or 5-hmC content according to the manufacturer's protocol. Assays were done in triplicate.

### Whole-mount *in situ* hybridization

Embryos that were collected and preserved in 100% methanol were processed for WMISH as previously described (Fong et al., 2014). The template for generating mouse *Tet1* riboprobes was a 1.8 kb cDNA containing part of the 3′ coding region (c.4333-6146) cloned into pBluescript (Stratagene, La Jolla, CA).

### RNA-Seq and statistical analysis

RNA was purified from the anterior part of E9 heads (18-22 somites) using the RNeasy Plus Universal mini kit (QIAGEN Inc., Valencia, CA). Quality and concentration was determined using a 2100 Bioanalyzer (Agilent Technologies) and NanoDrop (NanoDrop, Wilmington, DE). RNA with a RIN of at least 9 was pooled for a total of 300 ng from three biological replicates for each condition (wild type and mutant). RNA was poly-A selected using Dynabeads (Thermo Fisher Scientific, Waltham, MA). cDNA libraries were constructed using the Ion Total RNA-Seq Kit v2 (Thermo Fisher Scientific) according to the manufacturer's protocol for poly-A-selected RNA. RNA-Seq libraries were templated using an Ion OneTouch 2 System (ThermoFisher Scientific) and sequenced using a 200 bp kit on an Ion Proton sequencing system (Life Technologies) according to the manufacturer's instructions.

Ion Torrent Suite was used to obtain FASTQ sequencing data. Sequenced single-end reads (66,187,511 for wild type and 53,803,778 for *tuft*) were trimmed and filtered using PRINSEQ (Schmieder and Edwards, 2011). Low-quality sequences were trimmed from the ends until a base pair of Phred quality score ≥20 (at least 99% accurate) was not found, and filtered out sequences having below 20 nucleotides.

The *Mus musculus* UCSC mm10 reference genome was indexed by Bowtie2 v2.2.5. Processed reads were aligned to the reference genome using Tophat v2.0.14 (Kim et al., 2013). Tophat2 incorporates the Bowtie2 (Langmead and Salzberg, 2012) algorithm to perform the alignment. Resulting alignment (.BAM) files were analyzed with Cufflinks v2.1.1 (Trapnell et al., 2010), which quantified transcript abundance in terms of reads per kilobase of exon model per million mapped reads (RPKM). SAMtools v0.1.18 (Li et al., 2009) was used for sorting and BAM conversion, and htseq-count script on HTSeq package was used to count reads mapped to mouse gene models.

Differential gene expression from the count data was identified using the non-parametric NOISeq-sim program (Tarazona et al., 2011) with default parameters, a trimmed mean of M-values normalization and estimated probability of differential expression *P_NOI_* >0.95 as a threshold. The probability (1-*P_NOI_*) reported in NOISeq can be considered equivalent to q-value [false discovery rate (FDR)-adjusted *P*-value] (Zheng and Moriyama, 2013). Gene set enrichment analysis on the expressed genes was conducted using GSEA (http://www.broadinstitute.org/gsea/) with recommended default parameters of 1000 permutations and FDR<0.25 as a threshold for enrichment in phenotype. Data was deposited into the Gene Expression Omnibus (GEO), accession number GSE75001.

### Quantitative real-time PCR

RNA was purified using the Total RNA Plus Universal Kit (QIAGEN). 0.1-0.3 µg of RNA was used as a template for first-strand DNA synthesis (Bio-Rad Laboratories, Hercules, CA). Quantitative real-time polymerase chain reactions (qPCR) were performed using SYBR Green Universal Master Mix Dyes (Bio-Rad Laboratories), amplified and detected with the CFX96 Real-Time System C1000 Thermocycler (Bio-Rad Laboratories). Annealing temperatures and data capture for analysis was determined from melt curves and amplification efficiencies for each oligonucleotide primer pair (Integrated DNA Technologies, Coralville, IA) (Table S1). One microliter of first-strand DNA was used per reaction. Reactions were performed in triplicate for each biological replicate (*n*) and normalized using the 2^−ΔΔC(t)^ method (Livak and Schmittgen, 2001). Mean values of biological replicates were charted, with calculated standard deviation (s.d.) indicated by error bars. Samples with differences in expression levels were charted. Statistical significance (*P*) was determined by two-tailed, unpaired Student's *t*-test (R version 3.2.2) using ΔCt values to account for technical variance and sample size.

### Expression constructs and cell culture

Full-length clones of mouse *Tet1* mRNA (NM001253857.1, longer isoform 1) and one containing the c.5167C>T transition were assembled with gBlocks Gene Fragments (Integrated DNA Technologies) using the Gibson Assembly Method (New England BioLabs, Ipswich, MA). Sequences were confirmed followed by Maxi Prep purification (Sigma-Aldrich, St Louis, MO) prior to cell transfections. Mouse IMCD-3 cells [CRL-2123; American Type Culture Collection (ATCC), Manassas, VA] were transfected using the Gene Pulser Xcell electroporator (Bio-Rad Laboratories). Cells were seeded in six-well plates and cultured for 24-72 h in DMEM:F12 with 10% fetal bovine serum and antibiotics (Mediatech Inc., Manassas, VA). Media was replenished daily with or without 100 µg ml^−1^ of sodium L-ascorbate (Santa Cruz Biotechnology, Dallas, TX) following transfections. RNA or protein was harvested 24, 48 and 72 h following transfection. Transfected cells for immunostainings were seeded on sterile glass coverslips coated with PureCol EZ Gel Bovine Type I Collagen Solution (Advanced BioMatrix, San Diego, CA) diluted 1:6 following 20 min at room temperature. Cells were washed and fixed in 4% paraformaldehyde and permeabilized in 0.1% Triton X-100 (Sigma-Aldrich) prior to incubations with antibodies.

### Protein extraction and detection

Protein was extracted from tissue or cell cultures using the EpiSeeker Nuclear Protein Extraction Kit (Abcam, Cambridge, MA) according to the manufacturer's protocol. Protein concentrations were estimated against bovine serum albumin (BSA) standards (Thermo Fisher Scientific) using Bradford reagent (Bio-Rad Laboratories). 40 µg of protein were loaded per well of 4-15% gradient polyacrylamide gels (Bio-Rad Laboratories). Proteins were transferred onto Immobilon-FL nylon membranes (EMD Millipore, Billerica, MA) using Dunn's bicarbonate buffer with 0.1% sodium dodecyl sulfate. Proteins were detected using anti-TET1 specific for the N-terminus (Sigma-Aldrich, SAB2700188) or anti-polyhistidine epitope tag (anti-HIS; Cell Signaling Technology, Danvers, MA, mAb 27E8) diluted 1:1000 in phosphate-buffered saline (PBS) with 5% BSA overnight at 4°C. Bands were detected with secondary antibodies conjugated with IRDye (LI-COR Biosciences, Lincoln, NE) diluted 1:10,000 and scanned with the LI-COR Digital Imager (LI-COR Biosciences). Immunolocalizations were detected using goat anti-rabbit or mouse secondary antibodies conjugated with DyLight diluted 1:800 in PBS (Rockland Immunochemicals Inc., Limerick, PA) and counterstained with DAPI (Life Technologies) for nuclei. Images were taken at exposures <0.5 s based on negative controls using a DP73 microscope digital camera and cellSens Standard 1.12 software (Olympus Corporation, Tokyo, Japan).
